# Exploring transcriptomic diversity in muscle revealed that cellular signaling pathways mainly differentiate five Western porcine breeds

**DOI:** 10.1186/s12864-015-2259-9

**Published:** 2015-12-12

**Authors:** Magali SanCristobal, Florian Rohart, Christine Lascor, Marcel Bouffaud, Lidwine Trouilh, Pascal G.P. Martin, Yannick Lippi, Thierry Tribout, Thomas Faraut, Marie-José Mercat, Denis Milan, Laurence Liaubet

**Affiliations:** INRA, UMR1388 Génétique, Physiologie et Systèmes d’Elevage, F-31326 Castanet-Tolosan, France; Physiologie et Systèmes d’Elevage, Université de Toulouse INPT ENSAT, UMR1388 Génétique, F-31326 Castanet-Tolosan, France; Physiologie et Systèmes d’Elevage, Université de Toulouse INPT ENVT, UMR1388 Génétique, F-31076 Toulouse, France; Australian Institute for Bioengineering and Nanotechnology (AIBN), Corner College and Cooper Rds (Bldg 75), The University of Queensland, Brisbane Qld, 4072 Australia; INRA, UE450 Testage - porcs, F-35653 Le Rheu, France; Plateforme Transcriptome GeT-Biopuces, Laboratoire d’Ingénierie des Systèmes Biologiques et des Procédés (LISBP), F-31077 Toulouse, France; Plateau Transcriptomic impact of Xenobiotics (TRiX), ToxAlim INRA/INP, F-31027 Toulouse, France; INRA GABI, F-78351 Jouy-en-Josas cedex, France; IFIP/BIOPORC, F-35651 Le Rheu Cedex, France

## Abstract

**Background:**

Among transcriptomic studies, those comparing species or populations can increase our understanding of the impact of the evolutionary forces on the differentiation of populations. A particular situation is the one of short evolution time with breeds of a domesticated species that underwent strong selective pressures. In this study, the gene expression diversity across five pig breeds has been explored in muscle. Samples came from: 24 Duroc, 33 Landrace, 41 Large White dam line, 10 Large White sire line and 39 Piétrain. From these animals, 147 muscle samples obtained at slaughter were analyzed using the porcine Agilent 44 K v1 microarray.

**Results:**

A total of 12,358 genes were identified as expressed in muscle after normalization and 1,703 genes were declared differential for at least one breed (FDR < 0.001). The functional analysis highlighted that gene expression diversity is mainly linked to cellular signaling pathways such as the PI3K (phosphoinositide 3-kinase) pathway. The PI3K pathway is known to be involved in the control of development of the skeletal muscle mass by affecting extracellular matrix - receptor interactions, regulation of actin cytoskeleton pathways and some metabolic functions. This study also highlighted 228 spots (171 unique genes) that differentiate the breeds from each other. A common subgroup of 15 genes selected by three statistical methods was able to differentiate Duroc, Large White and Piétrain breeds.

**Conclusions:**

This study on transcriptomic differentiation across Western pig breeds highlighted a global picture: mainly signaling pathways were affected. This result is consistent with the selection objective of increasing muscle mass. These transcriptional changes may indicate selection pressure or simply breed differences which may be driven by human selection. Further work aiming at comparing genetic and transcriptomic diversities would further increase our understanding of the consequences of human impact on livestock species.

**Electronic supplementary material:**

The online version of this article (doi:10.1186/s12864-015-2259-9) contains supplementary material, which is available to authorized users.

## Background

After the study of genome evolution, much effort has been recently put on the evolution of gene regulation and gene expression (see Romero et al. [1] for a review). Several recent studies report the existence of differentially expressed genes across species. These differences in gene expression could be due to various evolutionary processes, neutral or not ([2–13]). All these studies focused on comparisons between species, with large evolutionary divergences.

For shorter evolutionary times, Hufford et al. [14] observed that candidate genes for domestication in maize do not display any specific expression profile, contrary to the genomic patterns (DNA sequence). For the domesticated period of sorghum, Jiang et al. [15] observed that gene expression divergence between two lines was mainly determined by DNA sequence divergence. Nätt et al. [16] on the contrary observed numerous gene expression and methylation changes between wild and domesticated chickens, with an overrepresentation in selective sweeps. For a shorter time scale, Yang et al. [17] used gene expression in addition to genomic polymorphism (SNPs) to assign a human individual to its ethnic population. Muller et al. [18] suggest that gene expression changes could be related to the out-of-Africa adaptation in Drosophila with a clear sex-specificity. In budding yeast, Fraser et al. [19] observed that entire pathways can be affected by adaptation of gene expression. Transcriptomic differences, as well as proteomics and metabolomics were shown between two genetically diverse dry bean germplasm by Mensack et al. [20].

As briefly shown above, some work has been conducted on the differences in gene expression across populations, these populations being mostly at species level. The following question is however less studied. Are there any differentially expressed genes across breeds of the same species? If yes, what are their biological functions? We propose to explore this problem in the particular context of a domesticated species with a long history of selection pressure from human: the pig. Perez-Enciso et al. [21] compared gene expression among pig breeds in several tissues and provided interesting results on gene expression divergence. However, only 16 animals were used in their study. The aim of our study was to see if genes were differentially expressed among the main Western pig breeds, at a larger sampling scale, and what might be the biological implications. Secondly, we aim to answer the questions what kind of gene expression characterizes a particular breed, and what differentiates a breed on the basis of gene expression. For that purpose, we will focus on one tissue of interest in pig breeding: a post-mortem muscle. Indeed pig meat production has placed a strong selective pressure on various characteristics of meat (muscle).

## Results

### Data

The sampled animals came from 5 pig breeds: 24 Duroc (DU), 33 Landrace (LR), 41 Large White dam line (LWF), 10 Large White sire line (LWM) and 39 Piétrain (PI). The two Large White lines derived from a common and recent ancestor, and were specialized on “male” traits (like conformation) for LWM or on “female” traits (like maternal behaviour) for LWF. All animals were males (castrates) except Piétrain (females). They were reared in 10 different contemporary groups, and slaughtered around 100 kg as in [22]. From these animals, 147 post-mortem *Longissimus dorsi* muscle samples were analyzed using the porcine Agilent 44 K v1 microarray. A total of 12,358 probes were detected and considered as expressed in muscle in the conditions of this experiment and after normalization. From these 12,358 probes, 9,055 (73 %) were mapped on the porcine genome (Sscrofa10.2 assembly) of which 8,758 (71 %) were localized on the autosomes.

### Global differential analysis

The aim was to identify genes whose expression varied across breeds. A large number of probes were found differentially expressed (DE). A list of 4,469 DE probes was identified between the five breeds for a FDR of 5 % (2,816, 1,703 and 1,095 for FDR of 1 %, 0.1 % and 0.01 % respectively; Table 1).

**Table 1:** Number of differentially expressed probes linked to the breed effect

FDR 0.1 %	Breed effect (global)	Pairwise	Global or pairwise	Global and pairwise	Only global	Only pairwise
All transcripts	1,703	1,655	1,858	1,500	203	155
Autosomes	1,228	1,232	1,374	1,086	143	146

Two statistical analyses were applied to the data: a Fisher test to identify genes whose expression, differ among the 5 breeds (Piétrain, Duroc, Landrace, Large White dam line, Large White sire line) and a pairwise comparison to identify which genes are differentially expressed between pairs of breeds. The table gives the number of differentially expressed probes for the analysis of all transcripts and for the analysis restricted to the transcripts localized on the genomic sequence of the autosomes

**Fig. 1:**
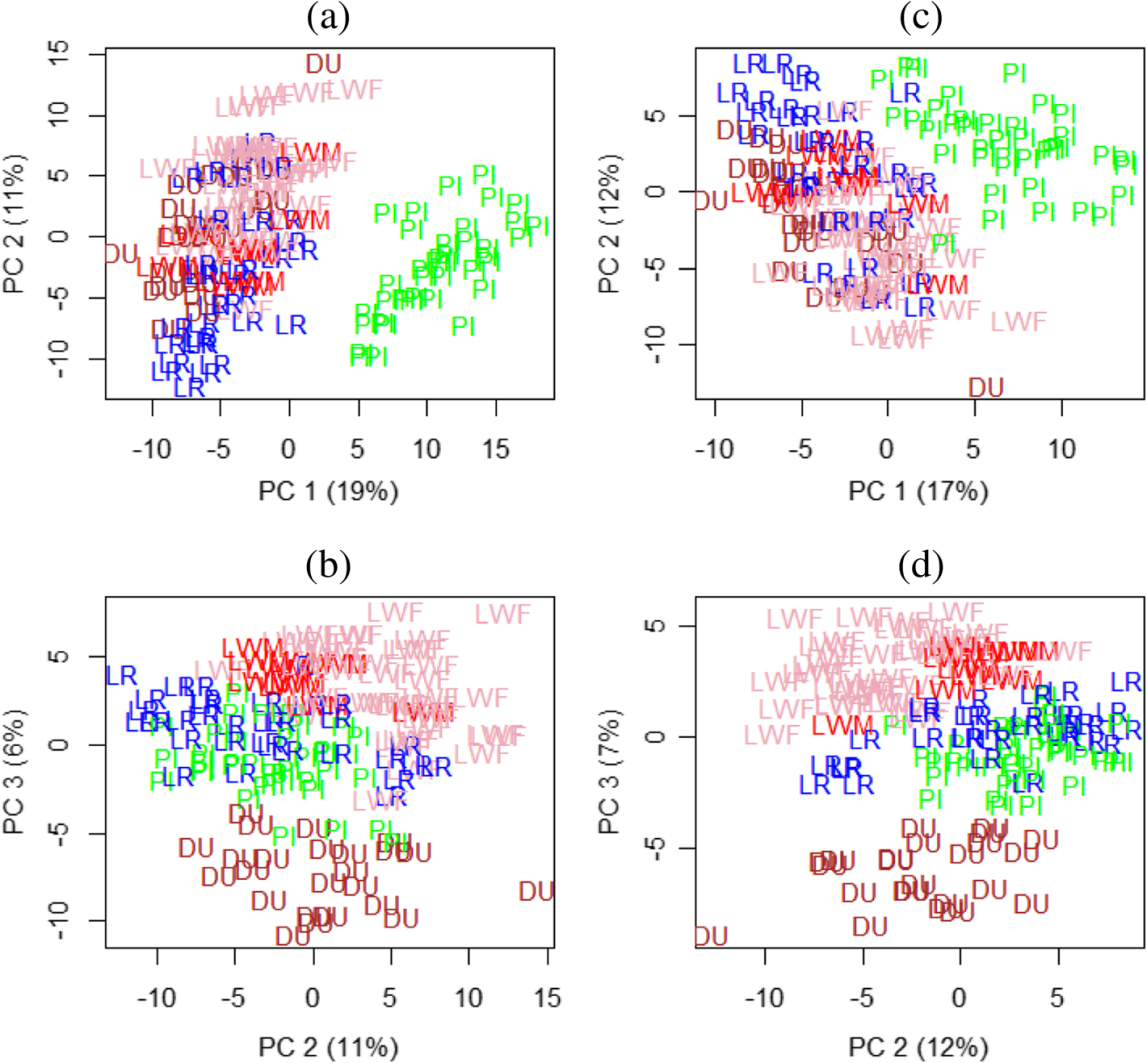
Principal Component Analysis of the top differentially expressed genes across breeds. Top 1,703 differentially expressed spots for a FDR equal to 0.1 % were used (**a** and **b**) and top 1,228 differentially expressed spots for a FDR 0.1 % restricted to genes localized on autosomes (**c** and **d**); (**a** and **c**) Principal Component (PC) 1 vs PC 2, (b and d) PC 2 vs PC 3. Piétrain (PI) animals are displayed in green, Landrace (LR) in blue, Duroc (DU) in brown, Large White sire line (LWM) in red and dam line (LWF) in pink

A principal component analysis (PCA) was performed on the 1,703 DE probes at 0.1 % FDR, or equivalently a Multidimensional Scaling on Euclidian distance between individuals. The first axis (PC1 on Fig. 1a) clearly separated Piétrain animals from the others with 19 % of the variability explained. Keeping in mind the confounding effect of sex and Piétrain, this result suggests that the sex effect was the most important effect on the variation of gene expression in post-mortem muscle of the main Western pig breeds. Although PC2 was difficult to interpret (no obvious known effect), PC3 separated Duroc from Large White animals (dam line and sire line together), with Landrace and Piétrain animals in between (Fig. 1b). PC4 and PC5 were able to separate Landrace from Piétrain animals (not shown).

**Fig. 2:**
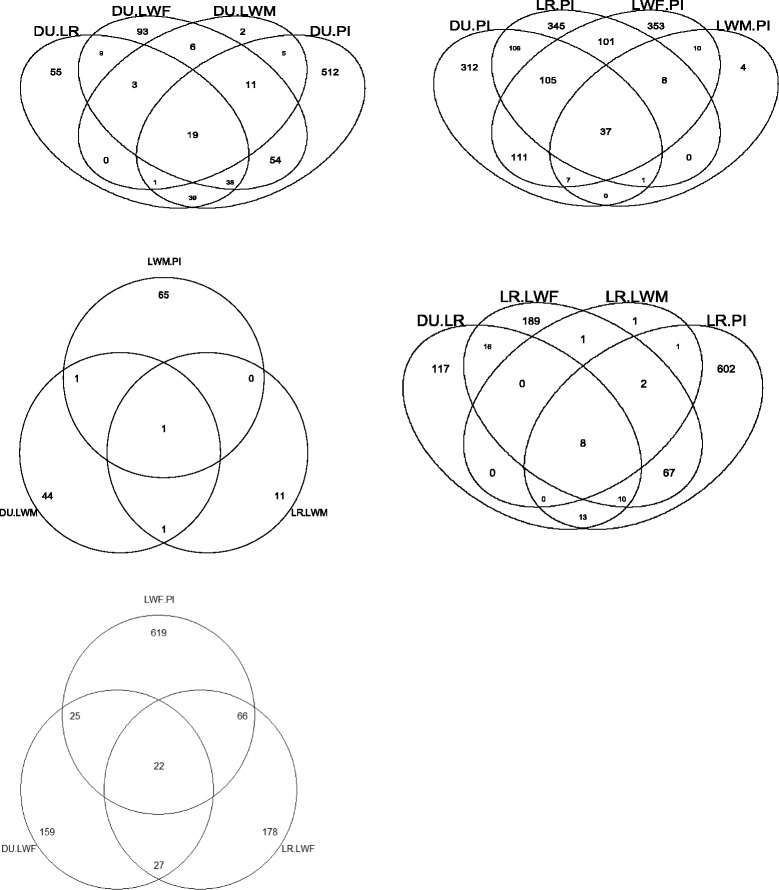
Venn diagrams of number of probes differentially expressed between breeds with pairwise comparisons. Comparisons between pairs of breeds were made at a FDR level equal to 0.1 %. The intersections between lists of differentially expressed probes are illustrated with Venn diagram, for each breed, to extract genes that are characteristic to one breed. DU: Duroc LWF: Large White dam line, LWM: Large White sire line, LR: Landrace, PI: Piétrain

In the differential expression analysis, tests of significance between pairs of breeds (pairwise analysis) allow the identification of 1,655 DE probes at a FDR of 0.1 %. A summary is given in Table 2. Although, most of these 1,655 DE probes were part of the 1,703 DE probes identified above by the global differential analysis, 155 DE probes were solely identified by the pairwise analysis (FDR < 0.8 % with the global analysis). A list of 1,858 DE probes, the union of global or pairwise analyses, was available for further functional analysis. The detailed statistical results for the 1,858 DE probes are listed in Additional file 1 with details of genes’ annotation on Additional file 2. As already seen on the PCA plots (Fig. 1a and b), Piétrain was the most different from the other breeds. Only six probes with a FDR 5 % appeared significant in the comparison of Large White lines, due to the small sample size in Large White sire line and the high genetic proximity of these lines (corresponding to five unique genes and one unannotated sequence; *NLRC5*, *CPNE2*, *RAD1*, *DHX33*, *PRKRIP1*, and *DN112586*). Venn diagrams for the other comparisons with a FDR of 0.1 % are plotted on Fig. 2.

**Table 2:** Number of differentially expressed probes between two breeds from the pairwise analysis

FDR 0.1 %	DU-LR	DU-LWF	DU-LWM	DU-PI	LR-LWF	LR-LWM	LR-PI	LWF-LWM	LWF-PI	LWM-PI
Global	164	233	47	641	293	13	703	6^a^	732	67
Autosomes	110	168	37	517	193	1	493	0^a^	519	40

^a^FDR 5 %

The number of differentially expressed probes between two breeds is given (FDR 0.1 %) for the analysis of all transcripts or the transcripts localized on the autosomes. Breed codes are DU for Duroc, LR for Landrace, LWF for Large White dam line, LWM for Large White sire line, and PI for Piétrain

**Fig. 3:**
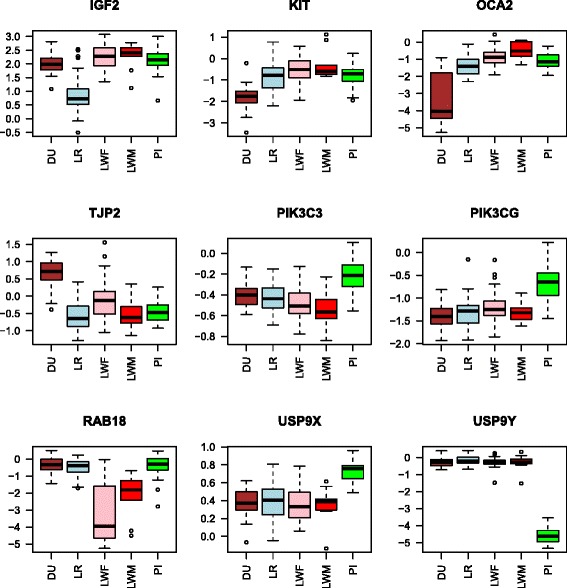
Examples of gene expression across the five breeds. The boxplots are given to illustrate the expression profiles of *IGF2*, *KIT*, *OCA2*, *TJP2*, *PIK3C3*, *PIK3CG*, *RAB18*, *USP9X* and *USP9Y* genes. The Y-axis represents the expression level (log transformed). Breeds are displayed as follows, Piétrain (PI) animals are displayed in green, Landrace (LR) in blue, Duroc (DU) in brown, Large White sire line (LWM) in red and dam line (LWF) in pink

Eight probes were detected as differentially expressed between Landrace and every other breed. Among them, seven probes corresponded to *IGF2* which is 3.4 fold decreased in Landrace compared to the other breeds (Fig. 3).

The overall intersect for comparisons with Duroc included the v-kit Hardy-Zuckerman 4 feline sarcoma viral oncogene homolog (*KIT*) [23] and oculocutaneous albinism II (*OCA2*) genes (among 19 probes in total corresponding to 17 unique genes and two unknown). *KIT* and *OCA2* genes are both mainly known to be involved in the determination of skin colour [24]. As expected, both genes are under-expressed in Duroc compared to the other pigs, white pigs or white spotted pigs (Fig. 3). This under-expression was observed in muscle tissue where *KIT* and *OCA2* encode membrane transporters and KIT is involved in the activation of the PI3K pathway.

Twenty-two genes were differentially expressed between the Large White dam line and the other breeds excluding the Large White sire line. The most differentially expressed gene is *RAB18*, a gene coding a small G proteins belonging to the Ras superfamily. This superfamily is known to coordinate vesicular trafficking in the cell [25]. Mutations in *RAB18* have been detected in humans with progressive neurological deterioration, and a severe hypotonia. Moreover, the variance of *RAB18* expression in skeletal muscle may suggest that this gene is especially genetically regulated in Large White dam line (Fig. 3).

Among the 37 genes differentially expressed between Piétrain and all the other breeds, 11 are localized on chromosomes X or Y. Two examples of gene expression are presented on Fig. 3 with the genes *USP9X* and *USP9Y*, respectively up and down regulated in Piétrain. This result corresponds to an over-representation of expression in females compared to males while a more widely held view is dosage compensation between XX and XY gene expression. In this work, as expected the *USP9* gene localized on Y chromosome was not expressed in female; while the expression of the *USP9* gene localized on X chromosome was overexpressed in Piétrain compared to its expression in the other breeds. Here where only Piétrains are female, we observed a doubled expression of the *USP9X* and no expression of *USP9Y*. The result would be an almost equal expression of USP9 proteins with probably similar function (assuming that both genes have exactly the same spatio-temporal expression profiles).

### Differential analysis restricted to the genes localized on autosomes

According to our preceding results and the description of how chromosome X is subject to selective pressure with more highly sex-biased gene expression [26], another differential analysis was conducted on the restricted list of 8,758 genes located on autosomes in order to lessen the sex effect observed with the first analysis. In these conditions, 1,228 DE probes were identified globally across breeds (FDR 0.1 %) and 1,232 DE probes for the pairwise comparisons (FDR 0.1 %). The union of both lists led to a total of 1,374 DE probes with a FDR for the global analysis of less than 0.8 %. These analyses (all probes and the ones restricted to autosomes) were done in parallel. That would allow the identification of the molecular basis of the difference between breeds including the Piétrain. However some genes (e.g. *IGF2*) were not localized because they are absent from the current assembly, even if their locations are well known (e.g. *IGF2* is on SSC2). Figure 1 (c and d) shows how avoiding X localized genes placed Piétrain pigs closer to the other breeds without changing the overall projection of the different breeds. The number of DE probes can be found in Tables 1 and 2. See Additional file 3 for details about probes on autosomes. No localized DE probes were identified between Large White sire and dam lines even with a FDR of 5 %.

Restricting the analysis to the autosomes is likely to be only a partial solution to the confounding effect of gender and breed. We chose to maintain Piétrain in the analysis because this breed is of prime importance for the breeders. Indeed, it has the highest muscularity and up to 80 % of the semen used in French production comes from Piétrain breed. Moreover, the gender effect may be reduced by the fact that all males were castrated in this work. We did the analysis without the Piétrain breed and the lists of DE probes were similar to those obtained with the Piétrain breed (not shown). The functional enrichment is hence similar for pairwise comparisons involving all breeds except the Piétrain.

### Functional annotation

Tables 1 and 2 underline the large number of gene lists to be analysed for biological interpretation. The functional analysis was undertaken in a sequential manner. First, the objective was to evaluate the impact of the sex effect of the Piétrain breed. While it would have been possible to restrict the functional analysis to autosomal genes, excluding important genes such as *IGF2* (absent from the current assembly) may give unreliable results. Therefore, the list of the 1,374 DE probes from the union of the global and pairwise comparisons restricted to autosomes was compared to the same list plus the 61 DE probes localised on chromosomes X and Y (Additional file 2). The free GeneCodis software [27] was used and the comparison was restricted to KEGG pathways, which are a collection of manually drawn pathway maps representing our knowledge on the molecular interaction and reaction networks. The top 10 significant pathways were exactly the same between both lists with or without genes on sex chromosomes (data not shown). According to the weak effect of the analysis restricted to autosomes on the PCA projection and on functional analysis, we hypothesize a low impact of the sex effect on our breed comparison, even if this effect could not be absolutely excluded.

The lists of DE genes from the union of global and pairwise analysis restricted (1,374 DE spots) or not (1,858 DE spots) to autosomes were compared through GeneCodis.

Whatever the input lists of DE genes to identify pathways significantly involved in the genetic expression diversity in muscle, the biological functions affected were almost the same (not shown). Finally, the functional analysis was restricted to the 1,703 DE probes (corresponding to 1,048 unique annotated genes recognized by GeneCodis) from the global analysis, and only KEGG pathways are presented. Seventy signaling and metabolic pathways were significantly enriched (FDR < 1 %). The most relevant pathways are presented in Table 3 and details of enriched pathways are summarized in Additional file 4. Among these seventy pathways, twenty-five included one PI3K gene (phosphoinositide-3-kinase; *PIK3C3* or *PIK3CG*). These pathways correspond to focal adhesion, regulation of actin cytoskeleton, interactions between cells or with the extracellular matrix (ECM); these pathways involved 82 genes.

**Table 3:** Relevant and significantly enriched biological functions (KEGG pathways) for diversity of muscle expression for five pig breeds

Items_Details	Number of genes	Adjusted *p*-Value^a^	Genes
Signaling pathways			
Focal adhesion	27	1.8E-08	*ITGA1*, *MAPK1*, *comP*, *ITGA6*, *ERBB2*, *LAMA2*, *CCND2*, *BCAR1*, *DIAPH1*, *PTEN*, *ITGA2*, *SPP1*, *ACTB*, *LAMC1*, *CAPN2*, *COL4A1*, *EGFR*, *VAV2*, *THBS4*, *ITGA9*, *THBS2*, *PTK2*, *PIK3CG*, *VEGFA*, *fn1*, *PPP1CB*, *VAV3*
Regulation of actin cytoskeleton	27	3.5E-08	*PIP4K2A*, *ITGA1*, *MAPK1*, *ARPC2*, *ITGA6*, *ITGB2*, *ARPC1B*, *F2R*, *BCAR1*, *DIAPH1*, *GNA12*, *ITGA2*, *NCKAP1L*, *GSN*, *ACTB*, *TMSB4X*, *EGFR*, *VAV2*, *ITGA9*, *CD14*, *PTK2*, *Kras*, *BAIAP2*, *PIK3CG*, *fn1*, *PPP1CB*, *VAV3*
ECM-receptor interaction	14	5.8E-06	*ITGA1*, *comP*, *ITGA6*, *LAMA2*, *ITGA2*, *GP1BB*, *SPP1*, *LAMC1*, *COL4A1*, *THBS4*, *ITGA9*, *THBS2*, *AGRN*, *fn1*
Adherens junction	12	2.7E-05	*MAPK1*, *PARD3*, *CTNNA3*, *ACP1*, *ERBB2*, *PTPRJ*, *ACTB*, *EGFR*, *BAIAP2*, *CREBBP*, *SORBS1*, *PVRL3*
Metabolic pathways			
Valine, leucine and isoleucine degradation	12	2.3E-07	*ALDH3A2*, *BCAT1*, *OXCT1*, *EHHADH*, *IVD*, *BCKDHB*, *ECHS1*, *ACADSB*, *HADHA*, *PCCB*, *AUH*, *ALDH2*
Lysine degradation	9	0.0002	*MLL5*, *ALDH3A2*, *EHHADH*, *NSD1*, *WHSC1L1*, *ECHS1*, *HADHA*, *setmar*, *ALDH2*
Purine metabolism	16	0.00033	*NUDT2*, *Pgm2*, *GMPR2*, *POLR2G*, *ENPP1*, *RRM2B*, *FHIT*, *GUCY1A3*, *ADA*, *NT5C2*, *NME6*, *PGM1*, *PRPS2*, *ATIC*, *NT5E*, *POLR3E*
beta-Alanine metabolism	6	0.00088	*ALDH3A2*, *EHHADH*, *ECHS1*, *CARNS1*, *HADHA*, *ALDH2*
Arginine and proline metabolism	8	0.0014	*ALDH3A2*, *ACY1*, *P4HA1*, *GLUD1*, *CARNS1*, *AMD1*, *ALDH4A1*, *ALDH2*
Fatty acid metabolism	7	0.0014	*ALDH3A2*, *EHHADH*, *ECHS1*, *ACADSB*, *ACSL4*, *HADHA*, *ALDH2*
Cellular processes			
Phagosome	21	9.2E-08	*comP*, *CTSS*, *ATP6V1C1*, *ITGB2*, *ITGA2*, *PIK3C3*, *ACTB*, *CYBB*, *CYBA*, *TFRC*, *THBS4*, *CD14*, *THBS2*, *NCF4*, *NCF2*, *DYNC1I2*, *EEA1*, *FCGR2B*, *MRC1*, *TUBA8*, *TUBA4A*
Protein processing in endoplasmic reticulum	21	7E-07	*nsfl1c*, *BAG2*, *UBE4B*, *PARK2*, *SAR1B*, *HSPH1*, *SEC24D*, *DNAJC1*, *CAPN2*, *DDOST*, *BCAP31*, *HSPA2*, *PDIA3*, *CUL1*, *UBE2D4*, *HERPUD1*, *EDEM2*, *DNAJA1*, *DERL1*, *CKAP4*, *TUSC3*
mRNA surveillance pathway	13	0.000016	*SAP18*, *PPP2R5A*, *CPSF6*, *CLP1*, *PPP2R2A*, *PAPOLA*, *PAPOLB*, *PPP2CB*, *Fip1l1*, *ETF1*, *HBS1L*, *CPSF3*, *CSTF2T*
Endocytosis	20	3.61E-05	*RAB11FIP5*, *PARD3*, *KIT*, *F2R*, *DNM2*, *EHD1*, *RABEP1*, *ARRB2*, *GRK5*, *DNM1L*, *FOLR1*, *EGFR*, *TFRC*, *HSPA2*, *WWP1*, *ZFYVE16*, *SMAP2*, *EEA1*, *epn1*, *FAM125B*

^a^Benjamini and Hochberg correction for multiple testing (FDR)

Top pathways obtained with the list of all differentially expressed genes using GeneCodis software, details are available in Additional file 4

### Breed discrimination

A differential expression analysis aims at giving a list of genes whose transcript abundances differ significantly among classes (here breeds). A discriminant analysis aims at identifying transcripts whose abundance differences help clustering (separating) each breed from the others. It helps predicting the breed to which a RNA sample belongs to, based on a list of “discriminant” genes. Although related, these 2 notions are different. In general a discriminant gene is a differential gene, but the reverse is not always true. Among a vast choice of discriminant methods, we performed a Random Forest (RF) analysis [28, 29] which is robust and non-linear and a sparse Partial Least Square – Discriminant Analysis (sPLS-DA) [30] that is pertaining to linear discriminators.

**Fig. 4:**
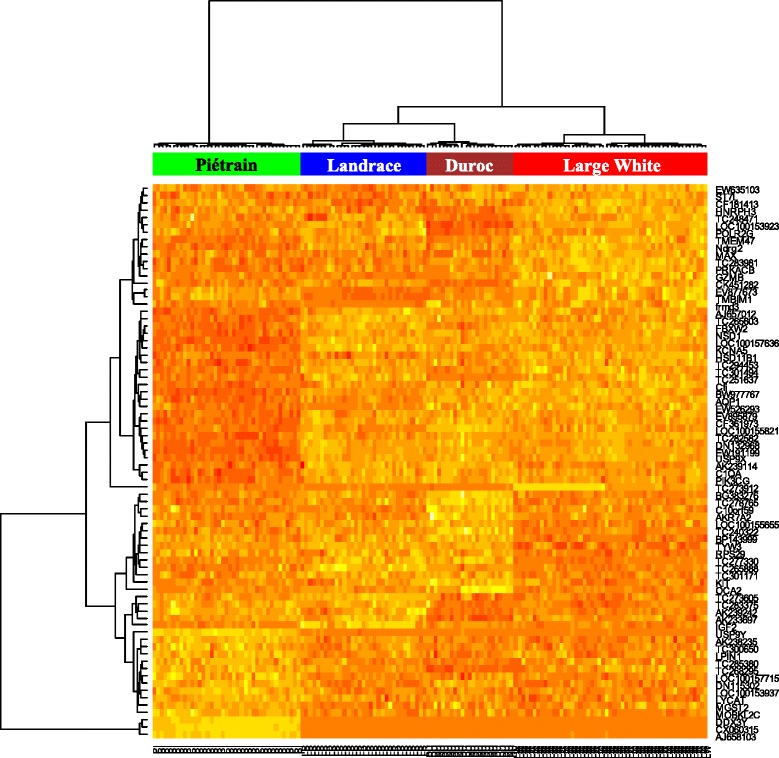
Heatmap of the annotated genes with the highest importance in breed prediction (Random Forest analysis). The 85 spots with highest Mean Decrease Accuracy importance correspond to 76 annotated genes. Piétrain (PI) animals are displayed in green, Landrace (LR) in blue, Duroc (DU) in brown, Large White (LW) in red

Firstly, it was impossible to determine whether a Large White sample originated from a Female line or a Male line with RF (not shown). Hence the two lines were merged in a single Large White (LW) breed in the following. We were interested in probe importance to identify stable predictors. The Mean Decrease Gini and Mean Decrease Accuracy importance gave almost identical results. The importance pertaining to each breed was also looked at to detect predictors for a particular breed. All the spots with the highest importance for a breed were included in a list of top 85 probes with the highest Mean Decrease Accuracy. These 85 probes corresponded to 76 unique gene names. To avoid an unbalanced weight on some genes due to the redundancy on the chip (e.g. *IGF2* was spotted 7 times and all *IGF2* spots were identified as important in the RF analysis), a heatmap with 76 genes (one probe for each unique gene name) was displayed, and provided in Fig. 4. The four breeds were perfectly separated with these 76 genes by a hierarchical clustering.

**Fig. 5:**
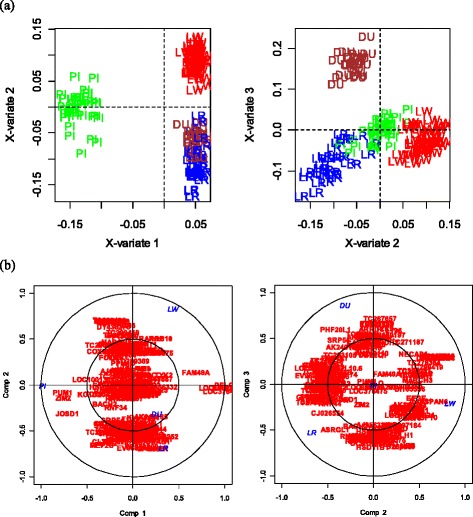
sPLS-DA with (8,100,70) spots selected on 3 dimensions. Plots of individuals (**a**) and correlations (**b**) between components and selected spots. Piétrain (PI) animals are displayed in green, Landrace (LR) in blue, Duroc (DU) in brown, Large White (LW) in red

Secondly, a sPLS-DA was conducted with three dimensions. A 10-fold cross validation led to a choice of 8 variables (probes) for the first dimension, around 100 for the second and 70 for the third dimensions. The first dimension separated Piétrain from the other breeds (Fig. 5a, similarly to the PCA on differential probes shown in Fig. 1). The second dimension allowed Landrace and Large White to be discriminated, while on the third and last dimension Duroc was opposed to the other breeds. The correlations of the probe loadings with the principal axes are given on Fig. 5b, and allowed a list of discriminant probes (then genes) to be extracted.

**Fig. 6:**
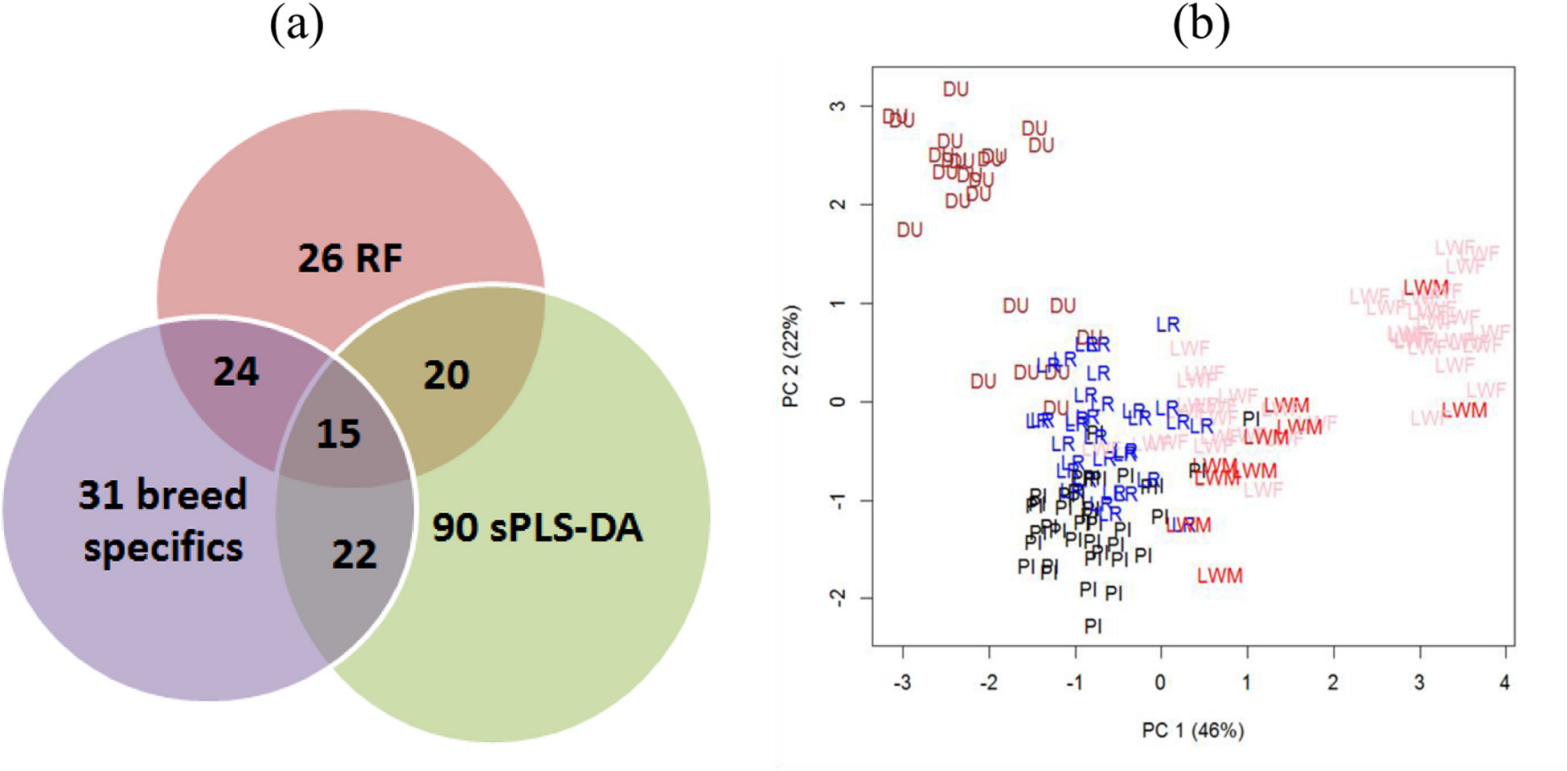
Three strategies to identify 15 genes allowing the discrimination of the porcine breeds. **a** Venn diagram with the 228 discriminant transcripts identified from one of three methods (Random Forest, sPLS-DA and genes differentially expressed between one breed and all the others); the 228 gene information is available in the Additional file 5. **b** PCA constructed with the 15 discriminant genes. The 15 discriminant genes are available in Table 4. DU: Duroc; LWF: Large White dam line; LWM: Large White sire line; LR: Landrace; PI: Piétrain

All these results are detailed in Additional file 5 combined with the DE probes which are differential from one breed compared to the others. The total list was composed of 228 probes that corresponded to 169 unique annotated genes. A short list of 15 genes was defined from the above three analysis (merging all the lists), and will be discussed in more depth in the following discussion (Table 4 and Fig. 6).

**Table 4:** Top 15 discriminant genes for breeds

Gene	Description	SSC	DEG breed-specific	sPLSDA-axis	Main functions
*KIT*	v-kit Hardy-zuckerman 4 fe line sarcoma viral oncogene homolog	8	DU	axis 2 (LW>DU,LR)	Endocytosis, cell growth and/or maintenance
*EMC2*	ER membrane protein complex sub unit 2	4	DU	axis 2 (LW>DU,LR)	Component of the ER membrane protein complex (EMC)
*TJP2*	tight junction protein 2 (zona occludens 2)	1	DU	axis 3 (DU>LR)	Tight junction
*PSMB4*	proteasome (prosome, macropain)sub unit, beta type, 4	4	DU	axis 2/3 (DU>LR,LW,PI)	Proteasome
*VDR*	vitamin D (1,25-dihydroxyvitamin D3) receptor	5	DU	axis 2/3 (DU<LR, LW, PI)	Mineral absorption
*OCA2*	oculocutaneous albinism II (pink-eye dilution homolog, mouse)	15	DU	axis 2/3 (DU<LR, LW, PI)	Transport of tyrosine
*RAB18*	RAB18, member RAS oncogene family	10	LWF	axis 2 (LW<DU,LR)	Two-component signal transduction system (phosphorelay)
*PRKACB*	protein kinase, cAMP-dependent, catalytic, beta	6	LWF	axis 2 (LW<DU,LR)	Protein amino acid phosphorylation; signal transduction; Insulin signaling pathway, Gap
*TW3*	tRNA-yW synthesizing protein 3 homolog (S. cerevisiae)	6	LWF	axis 2 (LW>DU,LR)	RNA translation, tRNA stabilization
*GZMB*	granzyme B (granzyme 2, cytotoxic T-lymphocyte-associated serine esterase 1)	7	LWF	axis 2 (LW<DU,LR)	Apoptosis; cytolysis; proteolysis and peptidolysis
*NAA20*	N(alpha)-acetyltransferase 20, NatBcatalytic subunit	17	PI	axis 1 (PI<DU,LR,LW)	CotranslationalN(alpha)-terminal acetylation of methionine residues
*MOB3C*	MOB kinase activation3C	6	PI	axis 1 (PI<DU,LR,LW)	Protein kinase essential for spindle pole body duplication and mitotic checkpoint regulation
*PIK3CG*	phosphoinositide-3-kinase, catalytic, gamma polypeptide	9	PI	axis 1 (PI>DU,LR,LW)	G-protein coupled receptorprotein signaling pathway; Regulation of actin cytoskeleton
*RNGIT*	RNA guanylyltransferase and 5’-phosphatase	1	PI	axis 1 (PI<DU,LR,LW)	mRNA capping; protein amino acid dephosphorylation
*DPHS*	diphthamide biosynthesis 5	4	PI	axis 1 (PI<DU,LR,LW)	Diphthamide synthesis pathway

From left to right: gene symbol, gene description, location on the pig genome (chromosome number), breed for which the gene is specifically differentially expressed, axis of the discriminant analysis (sPLS-DA) for which the gene has a great contribution, and main biological functions. *DU* Duroc, *LWF* large white dam line, *LR* landrace, *PI* Piétrain

## Discussion

The present work highlights the variability of the expression of about 1,800 transcripts between five pig breeds. These genes were identified for global differences (using a linear mixed model, 1,703 transcripts), and a pairwise analysis (1,655 transcripts). A subset of 228 transcripts was identified to be able to discriminate the five breeds in one way or another. A short list of 15, as the intersection of all lists, was extracted. Altogether, the union of all these highlighted transcripts corresponded to 1,230 unique genes.

### Variability of expression between breeds mainly concerns PI3Kinase signaling pathways involved in the regulation of muscle mass

**Fig. 7:**
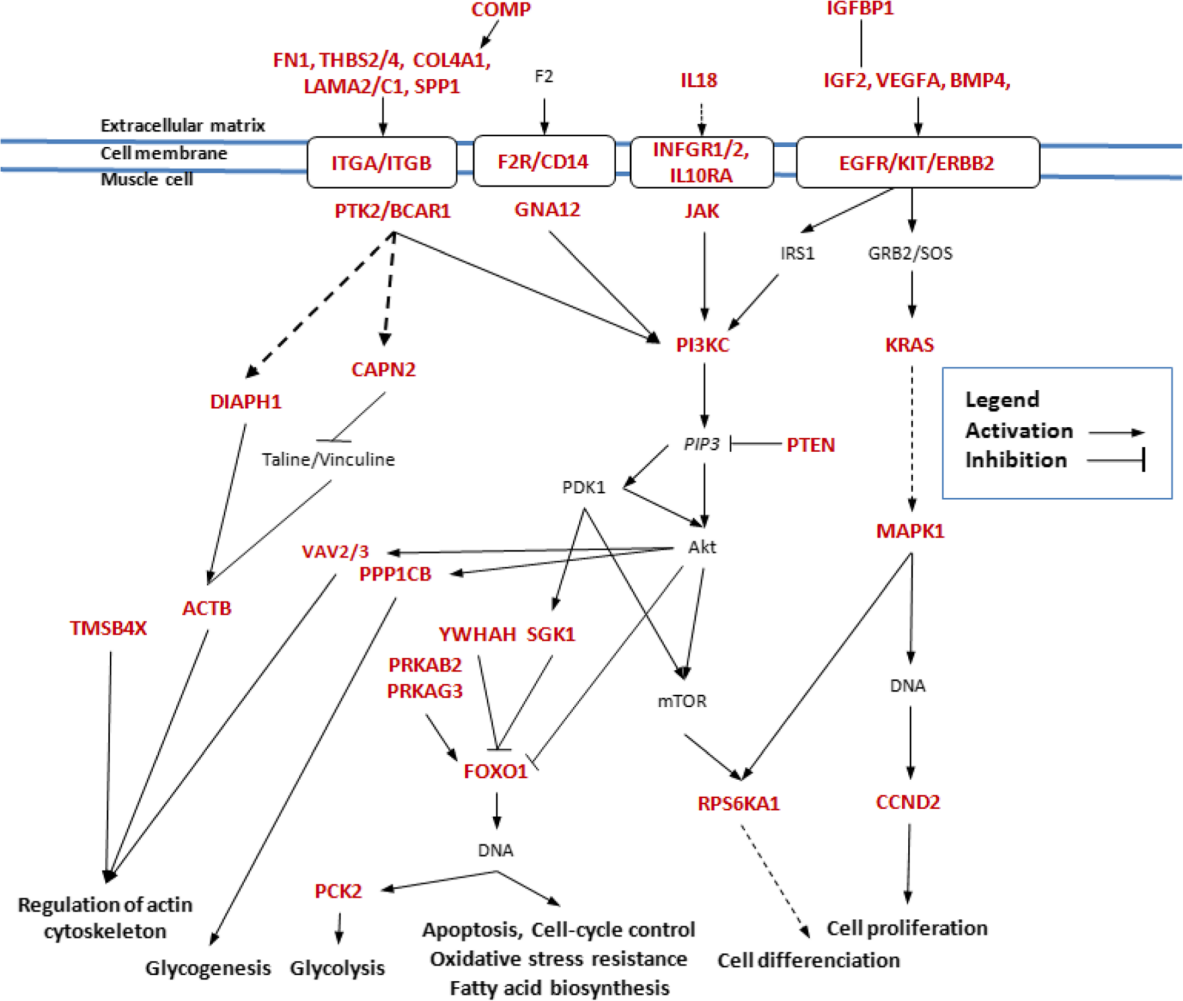
Schematic representation of the signaling pathways around PI3-kinase regulating muscle mass and metabolic processes. Genes in red are differentially expressed between the five porcine breeds whatever the direction (up or down) of the regulation of expression. This representation is a simplified summary of the corresponding KEGG pathways with only about 40 genes from the 82 involved

Biological functions enrichment analysis gave a large number of significant results. The most significant result is the huge number of genes involved in signaling pathways. Especially, the PI3 kinase pathways appeared to be the most relevant with 25 signaling pathways including PIK3C3 (3 pathways) or PIK3CG (22 pathways). A schematic and summarized pathway around the PI3 Kinase checkpoint was drawn (Fig. 7) and included only 40 out of the 82 involved genes. This diagram was constructed according to the description of regulation of muscle mass [31, 32].

Muscle mass is determined by both the number of muscle fibres and the size of these fibres. The development of muscle is temporally regulated and the total fibre number is fixed before birth (around day 90 of gestation, birth is around day 114) in pigs [33]. Afterwards, increase in muscle mass may be the consequence of mechanisms regulating muscle hypertrophy like the size of the fibres. In our study, muscle samples were collected at slaughter. Animals were about six months old and weighted around 110 kg. Therefore the expression analysis corresponds probably more to the hypertrophic process if existing. This hypothetical statement is in accordance with the results of the enrichment analysis that highlighted signaling pathways most regulated by PI3 kinase and known to be involved in the regulation of muscle mass. Then transcriptomic diversity between the five breeds underlined how these pathways regulating muscle mass may have been targeted by selection to increase lean meat production.

Upstream to the PI3K signaling pathway, regulated DE genes referred to interactions between cells or with the extracellular matrix (ECM) together with focal adhesion. These pathways are the three among the four top signaling pathways presented in Table 3 and in Fig. 7. DE genes products are presented at the ECM and cell membrane localization. This result is consistent with the importance of the ECM highlighted in muscle cattle in development [34]. As reviewed by [35], the extracellular matrix (ECM) is established to be a key player in muscle growth. ECM was most often described as an inactive component of cells. But many studies across species have now highlighted ECM functions, e.g. filtering, activating/inhibiting enzymatic activities, binding hormones, enzymes, and regulating the interaction of several ligands with their receptors. Most of the constituents of the ECM are mainly produced locally by the adjacent cells and most often by the fibroblasts present in the muscle tissue.

Also, PI3 kinase pathways regulate some energy metabolic pathways such as glycolysis and glycogenesis. Then selection on muscle mass may have also affected the regulation of these metabolic pathways. For example it has been suggested that decades of selection for more lean meat (more muscle mass) and larger litter size, may have increased piglet neonatal mortality [36]. As piglets have no brown adipose tissue, piglet thermoregulation at birth is essentially carried out by the skeletal muscle (shivering; [37]). It may be hypothesized that impaired glycolytic metabolism at birth, which is essential to ensure body thermoregulation, has been affected by genetic selection, affecting gene expression between breeds [37–39].

### Identification of some breeds specificities

#### 15 genes that discriminate duroc, Pietrain and large white

A total of 171 unique annotated genes were identified as able to differentiate the breeds. Among these 171 genes (from 228 probes), 15 genes were common in the two discriminant methods (Random Forest and sPLS-DA) and the pairwise differential analysis (Fig. 6). These 15 genes may be very interesting to better characterize the differences between the breeds. None of these genes were located on the sexual chromosome, which may imply they are less affected by the sex effect (confounding with the Piétrain breed) observed in this work.

#### Duroc

From these 15 genes, six genes differentiate the Duroc from the other breeds. Among the four genes under-expressed in Duroc, two genes are specifically known to be involved in skin coloration: *KIT* (v-kit Hardy-Zuckerman 4 feline sarcoma viral oncogene homolog or Dominant white locus in pigs; [40]) and *OCA2* (oculocutaneous albinism II). Both genes are coding transporter proteins and are involved in biological processes as transmembrane receptor protein tyrosine kinase signaling pathway for KIT and tyrosine transport for OCA2. *OCA2* gene was found over-expressed in white muscle (longissimus dorsi) compared to red muscle (soleus) in Meishan pig [41]. It is interesting to observe how genes related to skin colour and maybe submitted to selection may affect another tissue important for production traits. The term “melanocyte differentiation” was the first enriched gene ontology (q-value = 0.0043) with *KIT* and *OCA2* genes; Duroc pigs are brown-red.

Two genes are over-expressed in Duroc, *TJP2* (tight junction protein 2 or ZO2, zonula occludens-2; Fig. 3) and *PSMB4* (proteasome (prosome, macropain) subunit, beta type, 4). TJP2 is a membrane-associated guanylate kinase and encoded protein functions as a component of the tight junction barrier for cellular permeability involved in intercellular communication [42]. Moreover there is some evidence that at least another zonula occludens protein (ZO1) is involved in vascular remodelling processes [43]. These biological functions, cellular permeability and vascular remodelling, are interesting functions related to muscle meat traits, especially water holding capacity [44]. Similarly, the proteasomic proteins (as PSMB4) has a major role in degrading proteins in muscle cells [45] and the proteasome might be one of the endogenous proteolytic system contributing to meat texture development [46]. *PSMB4* with *VDR*, *KIT* and *OCA2* genes are involved in “reproduction process” (q-value = 0.0098). For example, the *VDR* (Vitamin D receptor) gene is known to be involved in male reproduction [47]. In Humans, it was observed to be down-regulated with leaner patient [48] and allelic variations in the *VDR* gene were identified to be associated with lean body mass and height in Human [49]. In French pig production the Duroc is used to obtain terminal boars.

#### Piétrain

In this study, five genes were identified to differentiate the Piétrain line from the others. Four genes were under-expressed in Piétrain compared to the others (*NAA20*, *MOB3C*, *RNGTT*, *DPH5*). The *NAA20* gene (N(alpha)-acetyltransferase 20, NatB catalytic subunit or *NAT5* gene) encodes a protein involved in normal cell proliferation and it functions in posttranslational protein N-terminal acetylation process [50]. Some specific proteins targeted by this N-terminal acetyltransferase were identified e.g. actin and tropomyosin; the human NatB complex depletion perturbs actin cytoskeleton and focal adhesion organization [51]. The *MOB3C* gene (MOB kinase activator 3C), member of the MOB protein family, has been shown to regulate mitosis, cell proliferation, apoptosis, centrosome biology and morphological changes [52]. The *RNGTT* gene (RNA guanylyltransferase and 5′-phosphatase) encodes a RNA guanylyltransferase involved in the regulation of gene expression with capping the 5′end of the mRNA (from NCBI/BioSystems). *RGNTT* was identified to be a potential candidate gene for average daily feed intake but not in Piétrain pigs [50]. The *DPH5* (diphthamide biosynthesis 5) gene is a component of the diphthamide synthesis pathway (from NCBI/Gene). This pathway regulates post-translational modifications [53]. None of these four genes are directly linked with the characteristic phenotypes of Piétrain (e.g. conformation). The fifth and up-regulated gene in Piétrain is *PIK3CG* (phosphatidylinositol-4,5-bisphosphate 3-kinase, catalytic subunit gamma; Fig. 3). *PIK3CG* is the only gene with a possible link with conformation as is one of the genes involved in the regulation of muscle mass (see first paragraph of discussion).

#### Large white sire and dam lines

The male and female Large White lines were difficult to differentiate from each other. Six probes corresponding to five unique annotated genes were found significantly different with a relatively low FDR (5 %). Among these five genes, four (*CPNE2*, *DHX33*, *NLRC5*, *RAD1*) were overexpressed in the male line and are coding for proteins with nuclear localizations. One of these genes was *NLRC5* (NLR family, CARD domain containing 5) and is the largest member of the NLR protein family (a NOD-like receptor). NLRC5 is an intracellular receptor involved in innate immune sensing; it is induced by interferons in case of pathogen infection [54, 55]. It is also involved in the regulation of kinase activity and NF-kappaB transcription factor activity (Biological Process Ontology from GeneCodis). The three other genes have mechanistic nuclear roles. *RAD1* codes a cell cycle checkpoint protein required for cell cycle arrest and DNA damage repair. *DHX33* (DEAH (Asp-Glu-Ala-His) box polypeptide 33) is identified to code a protein with an important role in rRNA transcription and cell proliferation. *CPNE2* (copine II) codes a calcium-dependent membrane-binding protein that may regulate molecular events at the interface of the cell membrane and cytoplasm. Only one gene, *PRKRIP1* (PRKR interacting protein 1 (IL11 inducible)) gene was over-expressed in Large White dam line. Little functional information is available for this gene except a negative regulation of protein kinase activity (Biological Process Ontology from GenoCodis) but it may play a role in cytokine-mediated biological functions [56].

#### Landrace

*IGF2* was among the few genes able to discriminate the Landrace from the others with random forest and the pairwise analysis (see boxplot on Fig. 3). One surprising point is that the sPLS-DA didn’t identify *IGF2* as a discriminant gene. The over-expression of *IGF2* in the other breeds is probably the consequence of the mutation in the *IGF2* intron3 g.3072G > A [57] which leads to an increased muscle mass and a reduced backfat deposition. This mutation has detrimental effects in prolificacy described as a result of an excess of leanness diminishing reproductive performance of the sow [58, 59]. In French breeds, the mutation favorable (allelic frequency of A > 96 %) to increase muscle mass seems fixed in Large White, Piétrain and Duroc, while the allelic frequency is always segregating in Landrace (about 70 % of allele A and 30 % of B; [60]).

The second gene identified as Landrace specific was *LBR* (lamin B receptor). *LBR* encodes an integral inner nuclear membrane protein. This protein is involved in the sequestration of heterochromatin near the periphery and the nucleoli in mammalian nuclei [61] and also in sterol metabolism [62].

### Transcriptomic diversity among breeds

Genetic diversity of Western pig breeds and lines has been studied over the past years using a range of genetic markers [63–67]. A clear separation of breeds was observed at markers that were specifically chosen to vary across breeds. On the transcriptome level, we observed here that breeds were also clearly separated on differential probes. The differential aspect of probes is equivalent to the pre-selection of genetic markers. In this study, after eliminating the confounding effect of sex and Piétrain, Duroc animals are the most distant to other breeds; that corresponds to the genetic basis and historical knowledge (Duroc came from America while Piétrain, Landrace and Large White originated from Europe). Then Piétrain and Large White are the most distant, again as with the genetic markers. So the overall picture of breed differentiation is most probably the same at the genomic and transcriptomic level.

Specific statistical tools are now able to detect signatures of selection at the transcriptomic level and compare them to the ones at the genomic level. This will be the focus of future work.

However, transcriptomic differences across breeds may reveal the impact of selection and help understanding genetic and phenotypic changes. Perez-Enciso et al. [21] also reported interesting patterns of differential gene expression across breeds in a study involving 16 animals per breed (Large White, Duroc, Iberian, and a cross with a Sino-European hybrid line). They observed hierarchical clusterings of breeds differing with tissues. Even if the five tissues were chosen from the endocrine axis (hypothalamus, adenohypophysis, thyroid gland, gonads and fat tissue), it is interesting to note that muscle differentiation was highlighted in their breeds’ transcriptomic differentiation.

More studies are needed to understand the effects of the various evolutionary forces (amongst which is selection) on the transcriptome of pig breeds.

## Conclusions

We found numerous differences in gene expression across the main pig Western breeds. Their functional analysis highlighted which biological function differed between these breeds and more precisely which genes from these functions differed between breeds. We hypothesized that these genes and related biological functions may have been targeted by human management (among which artificial selection) on production traits. The ideal situation would be that only terminal mechanisms (muscle mass, lipid metabolism…) for production traits vary across breeds. However our results showed that a multi-functional signaling pathway (PI3K) was affected. This pathway is well-known to be involved in the regulation of muscle mass and may impact the production traits that are selected for, but also “functional” traits around the regulation of metabolic pathway such as glycolysis. Moreover, most of the top discriminant genes between breeds were found to affect fundamental transcriptional and post-transcriptional mechanisms. These results could suggest how animal management and/or genetic selection may impact not only the terminal mechanisms of production traits but also the fundamental regulation of cellular processes such as regulation of gene expression and signaling pathways. Further analysis will help to decipher if robustness in farm animals was also impacted.

## Methods

### Animal sampling

All procedures and facilities were approved by French veterinary services (ethics committee: Direction Départementale de la Cohésion Sociale et de la Protection des Populations in Rennes, France; agreement number A35-240-7). All animals were raised at the French central test Station in Le Rheu (France) in 2007 and 2008, and slaughtered in the same commercial slaughterhouse (Cooperl-Hunaudaye, Montfort-sur-Meu, France). A total of 150 individuals were sampled, and came from 5 breeds: 24 Duroc (DU), 33 Landrace (LR), 41 Large White dam line (LWF), 10 Large White sire line (LWM) and 39 Piétrain (PI). These animals were castrates in all breeds except females in PI, were reared in 10 different contempory groups, slaughtered in 29 different series, each containing several breeds. Further details can be found in [22]. Muscle samples were biopsied from *Longissimus dorsi* (LD) muscle 20’ after stunning and exsanguination. Samples were immediately frozen in liquid N_2_ and kept at −80 °C until analysis.

### Total RNA extraction

The total RNA extraction was previously described in [68]. Total RNA was isolated from each of the 150 muscle samples. Briefly, the muscle samples were disrupted, homogenized and ground to a fine powder by rapid agitation for 1 min in a liquid-nitrogen cooled grinder with stainless steel beads. An aliquot of 250–300 mg of the fine powder was then processed for total RNA isolation and purification using RNeasy Fibrous Tissue Midi kit according to the manufacturer’s instructions (Qiagen SA France, Courtaboeuf, France). The method included a proteinase K digestion step to remove proteins and a DNase digestion step to remove contaminating DNA. The extracted total RNA was eluted in 300 μl of RNase-free water and stored at −80 °C. RNA quality and concentration were controlled using an AGILENT 2100 bioanalyzer (RNA solutions and RNA 6000 Nano Lab- Chip Kit, Agilent Technologies France, Massy, France).

### Microarray data: hybridization

The 4 x 44 K Porcine Gene Expression Microarray (G2514F, V1: 020109 Agilent Technologies; GEO accession number GPL10162) used in this work was previously described by the manufacturer. The 43,803 porcine probes sourced from UniGene (Release 33, Feb 2008), RefSeq (Release 27, Jan 2008), TIGR (Release 12, Jun 2006) using Agilent 60-mer SurePrint technology.

RNA concentrations were determined using a NanoDrop® ND-1000 spectrophotometer (NanoDrop Technologies, Wilmington, DE). cRNA labeled with fluorescent Cyanine 3-CTP was used for hybridization at 65 °C for 17 h onto porcine oligo microarray slides, according to the manufacturer’s recommendations (Low Input Quick Amp Labeling with Low Input QuickAmp Cy3 labeling kit One-color and One-Color Microarray-Based Gene Expression Analysis; Agilent Technologies). Hybridized microarray slides were scanned with scanner Genepix 4000B (AXON INSTRUMENTS) at 5-μm resolution. The scanned images were analyzed numerically using Agilent Feature Extraction Software version 9.5.3.1. (Agilent Technologies).

### Microarray data: normalization

The whole set of 45,220 spots were analysed on 181 chips (one sample per chip, a limited number of animals giving 2 to 3 samples). One chip was removed because of overall bad quality. Each spot on each chip was allocated a weight of 0 or 1 depending on various criteria of spot’s quality and signal to background level. On average, half of the spots per chip had a 0 weight. Only spots with a weight of 1 for at least 70 % of the chips were kept for further analysis, leading to 12,358 spots. All intensity data were log transformed, and the term “data” or “intensity” referred to log (natural logarithm) transformed data throughout the manuscript. The effect of the hybridization day was removed by subtracting the mean intensity of the hybridization day. Then the mean of each chip was subtracted to make all chips comparable.

A good linearity of intensity signals were observed between all samples. One animal was hybridized 3 times, and 32 individuals were sampled and hybridized twice. A very good adequacy was observed between samples from the same individual, with an average correlation of 0.98. Then, for each individual, only the sample with the best mean quality was kept. Finally, 147 chips representing 147 individuals were available for deeper statistical analysis. The normalized microarray dataset have been deposited in the NCBI Gene Expression Omnibus GSE56011.

### Differential analysis

A linear mixed model was fitted for each spot, with breed as fixed effect and contemporary groups as random effect, with the lme function of the R package nlme. The significance of the breed effect on gene expression was evaluated with an F-type test (R function anova). A False Discovery Rate (FDR, [69]) correction was performed on raw *p*-values (R function multtest). In the same linear mixed model, comparisons of each pair of breed were tested, with a FDR correction thereafter. In all cases, a threshold of 0.1 % has been applied except for the comparison between LWM and LWF (5 % threshold). This stringent threshold was chosen to ease the functional analysis; no enrichment can be highlighted with too many genes.

The fold change (FC) for any pair of breeds was calculated as the exponential of the difference between mean expression level in breed 1 and mean expression level in breed 2. This FC corresponds to the intensity ratio of mean raw signals on the microarray between breeds.

Hierarchical clusterings were built with the Euclidian distance and the Ward aggregation criterion for each clustering.

### Breed prediction

A Random Forest (RF) analysis was performed in order to predict the breed of each animal on the basis of its transcriptomic profile, using the random Forest package of the R software [70]. A preliminary RF analysis was used to eliminate the 70 % less important genes to avoid too much noise. On the basis of the remaining 30 %, i.e. 3,336 spots, a second RF analysis was performed with 5,000 trees (the other parameters were the default ones).

A sparse Partial Least Square – Discriminant Analysis (sPLS-DA) was conducted following [30], using the mixOmics R package (http://www.mixOmics.org).

### Probes annotation

The Agilent probes were annotated searching sequence homologies against following databases: SwissProt, TIGR Pig SsGI 12, UniGene Pig, Ensembl Human Transcripts NCBI36 (annotation from SIGENAE, http://www.sigenae.org/). The annotation of the DEG is summarized in Additional file 2. This file included the localization of the probes on Sus scrofa genome (Sscrofa10.2.69) and the human, bovine and mice orthologs were added when available (ortholog_one2one). Finally, some differentially expressed genes were manually annotated with blastn against the Refseq_RNA library (NCBI) or with blat against the pig genome (Ensembl, Sscrofa10.2 version).

### Functional annotation

From the Database for Annotation, Visualization and Integrated Discovery (DAVID), the software EASE (an Expression Analysis Systematic Explorer; https://david.ncifcrf.gov/ease/ease.jsp) was used to obtain functional Gene Ontology (GO) terms for each gene, the associated KEGG pathway, and the function summary. The systematic ontological annotation is given in Additional file 6.

In a second step, the GeneCodis website was used to identify co-occurrence in the functional annotation to highlight functions specifically enriched (http://genecodis.cnb.csic.es/; [27]). The Human genome was used as reference. We focused on KEGG pathways to avoid some redundant functional information. GeneCodis calculates an adjusted p-value. Most of the results for this work were obtained with GeneCodis with top pathways presented in Table 3 and details in Additional file 4.

## Availability of supporting data

The data set supporting the results of this article is available in the Gene Expression Omnibus (GEO) repository, with an accession number GSE56011, at http://www.ncbi.nlm.nih.gov/geo/query/acc.cgi?acc=GSE56011.

## Additional files

Additional file 1:
**1,859 genes were identified to be differentially expressed between five breeds according to statistical analysis.** 1,703 were differentially expressed with a linear mixed model (FDR < 0.1 %, column Pval. Breed.effect. BHcorrection) and 1,655 are declared differentially expressed between breeds with a pairwise analysis (FDR < 0.1 %; FDR < 5 % only for LWM compared to LWF, columns E to Y). This additional file corresponds to the analysis not restricted to autosomes. DU: Duroc; LWF: Large White dam line; LWM: Large White sire line; LR: Landrace; PI: Piétrain. (XLSX 515 kb) Additional file 2:
**Annotation of the regulated genes.** This list of probes corresponds to the differential probes from the linear mixed model, the pairwise analysis and the discriminating analysis. From this list, 1,394 probes were localized on porcine chromosomes (XLSX 650 kb) Additional file 3:
**Differentially expressed genes on autosomes.** Only probes with an alignment on the porcine genome Sscrofa10.2 are reported with the corresponding annotation from Ensembl. The global and pairwise statistics are given for all the comparisons. DU: Duroc; LWF: Large White dam line; LWM: Large sire Male line; LR: Landrace; PI: Piétrain (XLSX 689 kb) Additional file 4:
**70 pathways enriched to explain the biologically diversity of five pig breeds.** These pathways have been identified with the free software GeneCodis. These pathways concerned the KEGG (Kyoto Encyclopedia of Genes and Genomes) pathways which included the signaling and metabolic pathways. The pathways declared enriched have an adjusted p-value < 1 %. In the last column KEGG pathways including PI-kinases (PIK3C3 or PIK3CG) are highlighted. (XLSX 21 kb) Additional file 5:
**228 spots (171 unique genes) allow the discrimination between the five lines.** These genes were identified as differentially expressed from one line against the others or were identified in the sPLS-DA or Random Forest analysis. The 15 discriminant genes with the three methods are highlighted. DU: Duroc; LWF: Large White dam line; LWM: Large White sire line; LR: Landrace; PI: Piétrain. (XLSX 69 kb) Additional file 6:
**EASE systematic functional annotation for the 683 unique differentially expressed genes.** Detailed functional information were obtained from EASE (Expression Analysis Systematic Explorer) from DAVID (Database for Annotation, Visualization and Integrated Discovery) is given for each known gene when available: Official Gene Symbol, Gene identifiers (the human ortholog), Gene Name, Gene Ontology for Biological Process, Cellular Component and Molecular Function, KEGG pathway and a Function Summary (XLSX 178 kb)

## Abbreviations

CPNE2: copine II
DAVID: database for annotation, visualization and integrated discovery
DE: differentially expressed
DEG: differentially expressed genes
DHX33: DEAH (Asp-Glu-Ala-His) box polypeptide 33
DN112586: GenBank accession number for a non-annotated gene
DPH5: diphthamide biosynthesis 5
DU: duroc
EASE: expression analysis systematic explorer
ECM: extracellular matrix
FDR: false discovery rate
GO: gene ontology
IGF2: insulin-like growth factor 2
IPA: ingenuity pathways analysis
KEGG: kyoto encyclopedia of genes and genomes
KIT: V-kit hardy-zuckerman 4 feline sarcoma viral oncogene homolog
LR: landrace
LWF: large white: large white dam line
LWM: large white sire line
MOB3C: MOB kinase activator 3C
NAA20: N (Alpha)-acetyltransferase 20, NatB catalytic subunit
NLRC5: NOD-like receptor family CARD domain containing 5
OCA2: oculocutaneous albinism II
PCA: principal component analysis
PI: piétrain
PI3K: phosphoinositide 3-kinase
PIK3C3: phosphatidylinositol 3-kinase, catalytic subunit type 3
PIK3CG: phosphatidylinositol-4,5-bisphosphate 3-kinase, catalytic subunit gamma
PLS-DA: partial least squares discriminant analysis
PRKRIP1: PRKR interacting protein 1 (IL11 Inducible)
PSMB4: proteasome (prosome, macropain) subunit, beta type, 4
RAB18: RAB18, member RAS oncogene family
RAD1: checkpoint DNA exonuclease
RF: random forest
RNGTT: RNA guanylyltransferase and 5′-Phosphatase
sPLS-DA: sparse PLS-DA
SSC: sus scrofa chromosome
TJP2: tight junction protein 2 or ZO2, zonula occludens-2
USP9X or USP9Y: ubiquitin specific peptidase 9, x-linked or y-linked
VDR: vitamin D receptor
ZO1: zonula occludens
